# Label-Free Cyanobacteria Quantification Using a Microflow Cytometry Platform for Early Warning Detection and Characterization of Hazardous Cyanobacteria Blooms

**DOI:** 10.3390/mi14050965

**Published:** 2023-04-28

**Authors:** Yushan Zhang, Andres Escobar, Tianyi Guo, Chang-Qing Xu

**Affiliations:** 1 Department of Biomedical Engineering, McMaster University, Hamilton, ON L8S 4L8, Canada; 2 Forsee Instruments Ltd., Hamilton, ON L8P 0A1, Canada; 3 Department of Engineering Physics, McMaster University, Hamilton, ON L8S 4L8, Canada

**Keywords:** phycocyanin, cyanobacteria, microfluidics, fluorescence, microflow cytometer, *Microcystis aeruginosa*

## Abstract

The eutrophication of aquatic ecosystems caused by rapid human urbanization has led to an increased production of potentially hazardous bacterial populations, known as blooms. One of the most notorious forms of these aquatic blooms are cyanobacteria, which in sufficiently large quantities can pose a hazard to human health through ingestion or prolonged exposure. Currently, one of the greatest difficulties in regulating and monitoring these potential hazards is the early detection of cyanobacterial blooms, in real time. Therefore, this paper presents an integrated microflow cytometry platform for label-free phycocyanin fluorescence detection, which can be used for the rapid quantification of low-level cyanobacteria and provide early warning alerts for potential harmful cyanobacterial blooms. An automated cyanobacterial concentration and recovery system (ACCRS) was developed and optimized to reduce the assay volume, from 1000 mL to 1 mL, to act as a pre-concentrator and subsequently enhance the detection limit. The microflow cytometry platform utilizes an on-chip laser-facilitated detection to measure the in vivo fluorescence emitted from each individual cyanobacterial cell, as opposed to measuring overall fluorescence of the whole sample, potentially decreasing the detection limit. By applying transit time and amplitude thresholds, the proposed cyanobacteria detection method was verified by the traditional cell counting technique using a hemocytometer with an R^2^ value of 0.993. It was shown that the limit of quantification of this microflow cytometry platform can be as low as 5 cells/mL for *Microcystis aeruginosa*, 400-fold lower than the Alert Level 1 (2000 cells/mL) set by the World Health Organization (WHO). Furthermore, the decreased detection limit may facilitate the future characterization of cyanobacterial bloom formation to better provide authorities with ample time to take the appropriate actions to mitigate human risk from these potentially hazardous blooms.

## 1. Introduction

Continuously increasing human activities due to urbanization and population growth have led to excessive amounts of nitrogen and phosphorus in rivers, lakes, and oceans, otherwise known as eutrophication [1]. The resulting eutrophication has contributed to the increased reproductive rates of phytoplankton in aquatic ecosystems, leading to the accumulation of buoyant microorganisms that cause water discoloration and safety issues, known as blooms [1,2]. Within the varying species of phyla that can form a bloom, cyanobacteria are the most notorious and problematic as they have been increasingly found in water sources that have direct, or adjacent, relevance to the quality and safety of drinking water for urban environments [1,2,3,4]. Moreover, these blooms pose a further risk to the wildlife and urban life by depleting the water of valuable nutrients that may affect the delicate balance in each ecosystem surrounding these affected bodies of water. Cyanobacteria are a group of single-celled organisms that can sometimes produce toxins that are hazardous to both humans and other animals, otherwise known as cyanotoxins. The National Center for Environmental Health recorded data highlighting how 73% of bloom samples, in 7 states in the United States from 2007 to 2011, were found to be cyanobacteria [5]. The three most common cyanobacterial species were found to be *Anabaena* spp., *Aphanizomenon* spp., and *Microcystis* spp. [5]. The same investigation concluded that an overwhelming majority of the observed cyanotoxins (80%) were microcystins, a class of toxins produced by certain cyanobacteria [5]. Many of the observed microcystins are produced by *Microcystis aeruginosa* and can result in severe liver failure [6] and skin ailments [7] when consumed or with prolonged exposure [8]. *Microcystis aeruginosa* was, specifically, found to pose threats to recreational and drinking water sources through a study conducted in southern Quebec, Canada [9].

By improving our cyanobacterial monitoring and quantification capabilities, at all concentrations of cyanobacteria we may be able to mitigate the potential harmful effects caused by cyanobacterial blooms in critical sources of water. The World Health Organization (WHO) had previously developed a framework for the monitoring and management of a potential cyanobacterial bloom in various water sources, that would define concentrations of cyanobacteria at which the likeliness for bloom formation significantly increases [10]. The WHO have set Alert Level 1 and Alert Level 2 at 2000 cells/mL and 100,000 cells/mL, respectively, with the threshold for possible hazardous human effects set at 20,000 cells/mL [10,11]. It is worth noting that cyanobacterial cells can proliferate extremely fast in nutrient-rich environments at a warm temperature [11]. The literature on growth rates of eight cyanobacterial species highlights the doubling times of 1.65 days at 20 °C and 0.75 days at 29.2 °C [12,13]. The doubling times of cyanobacteria species, such as *Microcystis aeruginosa* and its doubling time of 1.23 days at 32 °C, can vary significantly across species, and in some cases can go well beyond 4 days [14,15]. As a result, these bacterial colonies may take days or months to form a bloom, making more generalized approaches to the management of bloom formation more difficult. Some of the most common strategies to prevent and control cyanobacterial blooms include nutrient management, hydrodynamics such as water diversion, and chemical and biological control [1,16]. Commonly used cyanobacterial bloom control methods may well be established but usually suffer from high costs, with their cheaper alternatives only offering temporary effects. Thus, it can be costly and quite difficult to control and reverse cyanobacterial blooms. Therefore, a label-free, rapid, and accurate detection and monitoring method for cyanobacteria at a level far lower than 2000 cells/mL is essential to provide early warning alerts to ensure there is enough time to respond to potential cyanobacterial blooms.

The best method is to stop potential blooms from growing as they begin to proliferate, in the earlier stages of development [16]. Conventional processes applied to drinking water treatment plants are not always fully capable of removing cyanobacterial biomass and cyanotoxins. To reduce the potential health impairments from cyanobacteria or cyanotoxins, it is quite important to stop potential cyanobacterial blooms at an early stage [17]. The monitoring of cyanobacteria in freshwater can be divided into three main method categories: biological, biochemical, and physicochemical [18]. Examples of biochemical and physicochemical methods include enzyme-linked immunosorbent assays (ELISA) and high-performance liquid chromatography (HPLC) [18]. These methods can often be more accurate, sensitive, and rapid, but require greater expense and skilled personnel for their use, hindering their feasibility for use. Instead, less expensive genomic and biological methods are more commonly employed. The most common alternative detection methods include biological microscopic-counting and genome-based indirect pigment measurement [18]. Laboratory-based quantification methods include microscopic quantification with a hemocytometer chamber, qPCR, and DNA-based chip assays. Hemocytometry is a commonly used biological method for cell counting that can be time-consuming and require trained personnel, similar to the genomic methods (e.g., qPCR assay, DNA-based chip) that have been successfully applied in the detection of *Microcystis aeruginosa*. Compared to biological assays, the biggest limitation of genomic assays is the complicated DNA extraction process. However, this complication can be avoided through the use of alternative methods such as indirect pigment detection [6,9].

Chlorophyll-a (i.e., Chl-a) and phycocyanin are two of the most commonly used photosynthetic pigments in the detection of cyanobacteria [10]. Detection depends on the positive correlation between the value of the pigments and cyanobacterial biomass such that pigment content in cells, such as Chl-a, is directly dependent on the density of total phytoplankton [10]. There are, however, several unique pigments that have a greater selectivity for cyanobacteria than others. One important example is the case of the pigment phycocyanin. Phycocyanin has a comparable fluorescence to Chl-a but has improved selectivity for cyanobacteria compared to a Chl-a-based detection method [11]. Phycocyanin-based detection measures in vivo fluorescence by transforming probes into a cell concentration of cyanobacteria [11]. Despite the improved selectivity of in vivo fluorescence probes such as phycocyanin, in early cyanobacterial detection they ignore the effects of the metabolic state and size of individual cells on pigment contents and can affect the phycocyanin reading between the true cell and hypothesized cell counts.

Several phycocyanin probes have been demonstrated to be capable of rapidly monitoring cyanobacterial-rich water samples, with varying levels of detection [11]. For the accurate detection of *Microcystis aeruginosa*, Bastien et al. reported a phycocyanin probe with a limit of detection of 4000 cells/mL [12], while another lab reported their phycocyanin probe to be 10,000 cells/mL [9]. However, these detection limits are insufficient to detect levels of cyanobacteria at the WHO Alert Level 1 threshold of 2000 cells/mL [13], and do not meet the WHO Alert Level 1 threshold required for cyanobacterial monitoring. Conversely, there exist commercial cyanobacterial probes with detection limits that meet the WHO Alert Level 1 threshold, but are limited to only providing qualitative results without a post-calibration correction for quantitative analysis, hindering real-time analysis [12]. Therefore, continuously increasing research effort has been made in raising the detection limit of these probes for cyanobacteria. One example, from Lee et al., demonstrated a quantitative detection method for *Microcystis aeruginosa* via NanoGene Assay, decreasing the detection limit to 9 agal cells/mL in water [6]. Unfortunately, methods with such low detection limits often have complicated preparation processes that can only be achieved in the laboratory. Therefore, microfluidic-based systems may offer an alternative which can address many of these limitations.

The microfluidic-based microflow cytometer is a powerful tool for cell identification and analysis, which can combine phycocyanin measurement with single-cell detection resolution [11,12]. Microflow cytometers employ side scatter and fluorescence-based detection methods, at low cost and small size, to facilitate both laboratory and on-site detection and quantification of cyanobacteria and other cells of interest [14]. Groups such as Hashemi et al. have successfully demonstrated the use of microflow cytometry for the optical analysis of phytoplankton at a minimum concentration of 50,000 cells/mL through data analysis using proper threshold setting and raw data filtering [14]. In addition, other groups have employed and further optimized similar data analysis methods for particle/cell monitoring, at single-cell resolution, through microfluidic devices [15]. Therefore, this paper will propose a detection and quantification method suitable for on-site monitoring of *Microcystis aeruginosa*. The paper proposes and demonstrates, based on side scatter and fluorescence of phycocyanin using a microflow cytometer, improvements crucial to on-site monitoring of cyanobacteria such as reduced cost, label-free probing, increased accuracy and lowered testing time.

First, the performance of the microflow cytometer on *Microcystis aeruginosa* detection and quantification was investigated. By employing both amplitude and transition-based thresholds, fast (<1 min) detection and quantification of *Microcystis aeruginosa* was achieved. With the ability to achieve a relatively low detection limit of ~10,000 cells/mL, sufficient to identify concentrations below the WHO Alert Level 1 threshold of 2000 cells/mL, this platform can be used to characterize the potential for early-stage cyanobacterial blooms [16]. Moreover, through the addition of a pre-filtration process, a novel detection limit as low as 5 cells/mL was achieved. Improving the detection limit is extremely important in early characterization of the potential signs of potentially hazardous cyanobacterial blooms and potentially provides public health workers with more time to better inform and protect the public.

## 2. Materials and Methods

### 2.1. Developing the On-Chip Microflow Cytometer for Cyanobacterial Detection and Quantification

**Figure 1:**
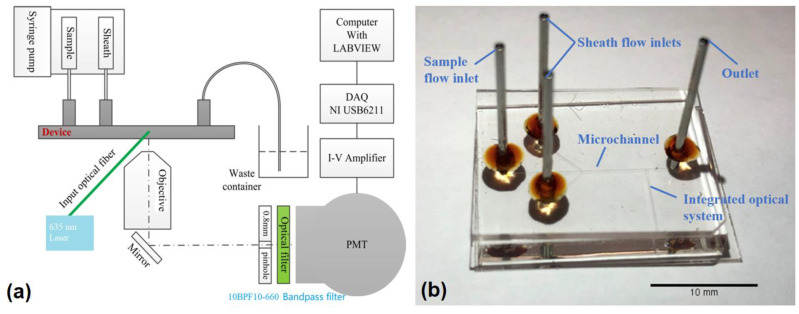
(**a**) A schematic diagram of a microflow cytometer. (**b**) A photo of a microfluidic device with integrated optics.

The development of a microflow cytometer fundamentally relies on the integration of on-chip waveguides and micro-lenses with a microfluidic platform (Figure 1) to be able to process samples at a high volume [17]. Figure 1b depicts the microfluidic device, fabricated with photolithography, that is used in this study. The three main components of this device, from top to bottom, consist of a glass layer for protection, a polydimethylsiloxane (PDMS) layer for sealing, and a glass substrate coated with a functionalized SU-8 containing the microchannel system. The 100 µm wide and 50 µm height microchannels were fabricated by functionalizing SU-8 onto a glass substate via UV exposure, using a photomask containing the entire microchannel design. This photolithography process has been studied and optimized multiple times in previous publications [17,18,19,20,21,22,23].

The sample of interest was hydrodynamically focused to the center of the channel by sheath fluids running along the sides of the channel. This hydrodynamic focus facilitates single-cell resolution and quantification by maximizing the likeliness that only one cyanobacterial cell passes through the optical system junction in any single instance. As a result, the detectable light-scattering interactions between the generated light and cells of interest could be, and were, observed. A generated laser light, with a wavelength of 635 nm, was directed by the on-chip waveguides to excite the cells of interest and induce scattered light and fluorescence events through cyanobacterial in vivo fluorescence. The scattered light was deflected by mirrors to a photomultiplier tube (PMT) fitted with a 0.8 mm pinhole and a 660 ± 10 nm bandpass filter to reduce unwanted background noise and side-scattered light. The raw data was amplified and converted into voltages before being further processed by a data acquisition card (DAQ) purchased from National Instruments (NI). Customized LabView programs were used in determining the proper gating thresholds for pulsed data and to quantify the presence of cyanobacterial samples. This platform ensures that no labelling method is required in the detection platform, enhancing its potential as an on-site tool for cyanobacterial monitoring and quantification.

### 2.2. Microcystis aeruginosa Preparation

*Microcystis aeruginosa* samples from the Canadian Phycological Culture Centre (CPCC) used in these experiments were grown and cultured in 3N BBM medium glass tubes. A two-fold serial dilution was performed in 10 mL tubes to reduce the cell abundance of highly concentrated cyanobacterial samples (10^7^~10^8^ cyanobacterial cells/mL), then further resuspended in phosphate-buffered saline (PBS) prior to measurement. By using a hemocytometer chamber and a microscope, the cell counts for the cultured cyanobacteria was measured, at least in triplicates, and found to have a cell count variance of less than 10%.

### 2.3. Pre-Filtration and Concentration Device for Enhanced Recovery of Microcystis aeruginosa

Through the addition of a pre-filtration and concentration step prior to analysis, the on-site detection and quantification potential of the microflow cytometer to effectively analyze low-level cyanobacterial concentrations could be significantly enhanced. Thus, a paired sample treatment system was developed to facilitate the processing of large sample volumes (>1 L) in a shorter time.

#### 2.3.1. Prototyping the Automated Cyanobacterial Concentration and Recovery System (ACCRS)

**Figure 2:**
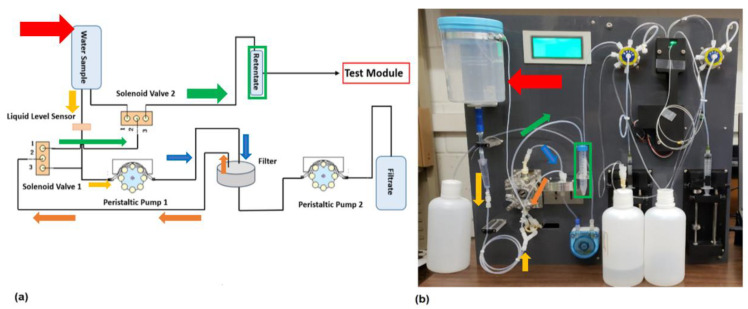
Automated cyanobacterial concentration and recovery system (ACCRS). (**a**) A schematic diagram of the ACCRS, (**b**) a picture of the physical setup. Order of flow (for sample of interest) follows directions: red, yellow, blue, orange, green.

Figure 2 demonstrates both the working principle (Figure 2a) and the prototype device (Figure 2b) of the proposed automated cyanobacterial concentration and recovery system (ACCRS). The large volume water sample is fed through the system by a primary peristaltic pump (Pump 1), until it reaches the chamber housing a ceramic filter of a 0.14 µm pore size. As *Microcystis aeruginosa* are often larger than the pore size, the cells of interest remained on the surface of the filter membrane, while water molecules and particles smaller than 0.14 µm ran through the filter. This filtrate, containing molecules smaller than 0.14 µm, was subsequently pumped into a filtrate reservoir by a lower powered Peristaltic Pump 2. As Peristaltic Pump 2 filters out the particles smaller than 0.14 µm, the larger cyanobacteria left on the ceramic filter is then flushed out of the chamber by back-flushing towards Solenoid Valve 1. The negative pressure generated by Peristaltic Pump 1 will help flush the cyanobacteria that is fixed on the ceramic filter through to Solenoid Valve 1 via tangential flow. Once at Solenoid Valve 1, the incoming fluid from the filter chamber will enter at Solenoid Valve 1-Port 2 (SV1-P2), before being evenly distributed between Solenoid Valve 1-Port 1 (SV1-P1) and Port 3 (SV1-P3). The fluid that exits through SV1-P1 will direct the fluid to Solenoid Valve 2 (SV2) and will direct it towards the Retentate reservoir, where the concentrated solution of cyanobacteria will be collected. The fluid that is not directed towards SV2 will be redirected back towards Peristaltic Pump 1 to repeat the process. This entire process will continue until either the initial water sample runs out, or when the final retentate has reached its desired volume, measured by the liquid level sensor. Once the final milliliter volume of retentate has been reached, the cyanobacterial cells further resuspended in microliter volumes of PBS for introduction to the microfluidic device. The resulting device was coined the automated cyanobacterial concentration and recovery system (ACCRS), as shown in Figure 2.

A similar setup has been created and applied for the pre-filtration of *E. coli*, and the detailed working principle and detailed experimental data can be found in previous publications for concentrating and recovering *E. coli* [15,24]. To meet the requirements of cyanobacterial pre-filtration, based on the previous and commonly cited literature, a new setup was established based on previous experimental results and empirical analysis [24]. A tangential flow technique and unique back-flushing techniques were the main methods used to improve the recovery efficiency of the system. As a result, the parameters of the pumping tube and back-flushing techniques were optimized by fine-tuning the parameters of the two main pumps. Most importantly, the final retentate volume was reduced significantly from ~5 mL to ~1 mL by a systematic study of controller parameter optimization and hardware enhancements.

To calibrate and assess the recovery rate, several known amounts of *M. aeruginosa* were spiked into 1000 mL PBS before being run through the ACCRS. It was successful in increasing the total concentration of the samples to be tested by the microfluidic platform, while simultaneously decreasing the assay volume required for accurate bacterial detection. This was determined by comparing the quantitative and qualitative analysis results of control groups that were not processed by the ACCRS against sample retentates that were processed by the ACCRS in the microflow cytometer. In comparison to several other commonly used pre-filtration steps, such as filter membranes and centrifugal-based methods, this filtration and concentration setup significantly improves recovery rates of cyanobacteria through the implementation of a back-flushing system [25]. It does not fall victim to the same type of inherent cost limitations as filter membranes due to its use of a reusable ceramic filter membrane, which can be used many times before requiring a cleaning step. Thus, reducing the cost of purchasing one membrane filter for every individual test, as well as preventing filter membrane clogging, is commonly observed in conventional filter membranes [23]. This back-flushing system minimizes the amount of uncontrolled cyanobacterial cell rupturing and cell resuspension that would reduce the quality of the recovered cells. The gas vesicle rupturing observed in many planktonic cyanobacteria, including *Microcystis*, has been directly correlated to lowered recovery rates observed in many conventional centrifugation-based biomass enrichment techniques [25]. Therefore, this microflow cytometry-integrated pre-filtration system presents a novel and practical means to maintain heightened cyanobacterial recovery rates and further facilitates a decreased detection limit of cyanobacteria, such as *Microcystis.*

#### 2.3.2. Updated Automated Cyanobacterial Concentration and Recovery System (ACCRS)

In the original design of the ACCRS prototype, as in Figure 2b, the main focuses were to improve the overall recovery rate of cyanobacteria by adding a pre-concentration and filtration step that would help to reduce the sample volume and remove undesired molecules from the samples. Once this proof of concept was achieved, to optimize the use and on-site potential of this system, later efforts to improve the overall user experience and facilitate the testing and management of the platform became paramount. Thus, the subsequent reduction of the overall device size (Appendix A), the integration of a pre-programmed concentration and cleaning protocol, and the reduction of the sample assay volume have significantly improved the overall user experience. As a result, the proposed platform is making it much more practical for on-site detection of cyanobacteria.

## 3. Results and Discussion

### 3.1. Fluorescent Results of Microcystis aeruginosa

**Figure 3:**
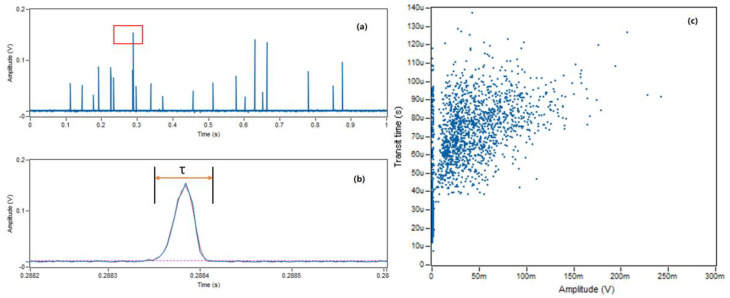
Raw data from microcystis aeruginosa fluorescent measurements. (**a**) Fluorescent signals of one second from one experimental measurement. (**b**) The enlarged view of one pulse in (**a**). (**c**) All events plotted with extracted transit time and amplitude in the same measurement with the amplitude and transit time thresholds defined by the box of 5–150 mV and 35–115 µs.

The light scattering events were captured and recorded using a customized LabView program by demonstrating the amplitude and transit time of the cyanobacteria passing through the detection region. A one-second readout from the recorded *M. aeruginosa* samples, using the microflow cytometer at a particular flow rate of 2000 µL/h, is shown in Figure 3a. It is important to note that our flow rates can vary from 2000 µL/h to 10,000 µL/h, and also vary depending on the sample volume being used, to ensure the entire experiment can be completed in under an hour. The individual peaks are representative of the magnitude of the light scattering event the cyanobacterial cells produced, as a measure of each instance of induced in vivo fluorescence by individual cyanobacterium passing through the detector [26,27,28]. The spacing between individual peaks suggests that the proposed single-cell resolution of this platform was maintained consistently, as there were very few instances in this one-second readout where multiple peaks were overlapping. Thus, each individual peak is representative of a single cell passing through the laser at any given time, with its respective fluorescence signal intensity (in millivolts) being proportional to the size of the cell. Overlapping peaks would show on the plot of Figure 3a as broad, thicker peaks that suggest more than one cyanobacterial cell was detected at almost the exact same time. In the collected data seen in Figure 3a, each peak only represents the macro-perspective of a cell passing through and being detected by the device. Each individual peak can, however, be more clearly characterized by their picosecond behavior, shown in Figure 3b. The peak highlighted in the red box is enlarged and demonstrates their signals as quasi half-sine pulses with specific amplitudes (y-axis) and transit times (τ). The amplitude and transit time of each cyanobacterium passing through the detector may elucidate behavior of the cyanobacteria under investigation that could only be shown by plotting their transit times against the amplitude of the signal. The relationship between transit times and amplitudes within this detection platform, taken over 60 s, is presented in Figure 3c. The collective of peaks that appear in a line on Figure 3c show little amplitude and may not be wholly representative of a *Microcystis aeruginosa* cyanobacterium, as they appear to be outliers. Thus, thresholds and standardized tests must also be performed to improve the quality of information we can infer from the data.

It can be seen in Figure 3c, captured by the yellow square that the majority of data points fall within the 35 µs to 115 µs range, and within 5–150 mV. These threshold values were used in subsequent tests to more rigorously distinguish positive signals from background noise. Due to the 2D hydrodynamic focusing used to enhance the resolution of the device, the actual transit time appears to have a much larger range than the theoretical calculated transit time of 63–81 µs. In conclusion, a transit time threshold can be applied to improve the accuracy of the measurement of side scattering detection of particles and will help determine a standardized amplitude and transit time for true positive signals of *Microcystis aeruginosa* [17].

### 3.2. Validation Measurements for Optimal Microflow Cytometry Use

**Figure 4:**
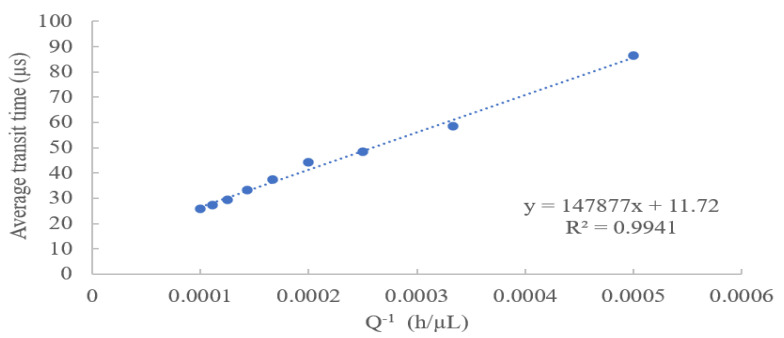
Relationship between measured average transit time and the inverse of total flow rate.

To ensure that the flow rate and average transit time are not subject to other unsuspecting variables, a comparison of average transit time to the inverse flow rate was performed. *Microcystis aeruginosa* cyanobacteria was pumped through the microflow cytometer at total flow rates between 2000 µL/h to 10,000 µL/h, under a sample to sheath fluid flow rate of 1:3, respectively. Figure 4 demonstrates the observed inversely linear relationship between the total fluid flow rate and the average transit time. Specifically, the average transit time linearly decreased from 86.34 µs to 25.93 µs when changing the total flow rate from 2000 µL/h to 10,000 µL/h. In addition, with a good inversely linear (R^2^ = 0.9941) relationship between the average transit time and the total flow rate, the minimum threshold used to distinguish outliers also changed. At the upper limits of the flow rate the threshold was 15 µs, while at the lower limit of the flow rate the transit time threshold was set to 40 µs. This information suggests the thresholds for transit time are dependent of the total flow rate used in the system and justify their use as effective tools to determine outliers in data analysis related to microflow cytometers. The good linearity also indicates that the microflow cytometer is stable under various experimental conditions and minimizes environmental interference of data collection.

Each of the *M. aeruginosa* samples were measured multiple times under different conditions by the same microflow cytometer and the results are shown in Table 1. Table 1 is used to highlight the variance in measurements of replicate samples to demonstrate the ability of this technique to be used at varying cyanobacterial concentrations. The sample cell concentrations were prepared by serial dilution and ranged from 10^4^–10^6^ cells/mL. The coefficient of variation (CV) data highlighted in Table 1 demonstrates a relatively large variation in concentrations at lower average concentrations, compared to the higher concentrations. Despite the CV of many cell concentrations falling within 10%, there were still two samples that had a significantly high variance, which may suggest the need for greater improvement of the microflow cytometer at lower concentrations. The increased CV of low concentration samples, such as sample 6 and 7, can be attributed to slight deviations in the quantification of the cyanobacteria. Any slight outlier would then skew the standard deviations of each sample and increase their CVs. The quantitative measurement of *M. aeruginosa* by the microflow cytometer is reliable across a wide range of cyanobacterial concentrations. Moreover, the significance of having the means to measure these wide concentration ranges was highlighted by the concentration device, as the previous limit of detection without the concentration device was 10,000 cells/mL. 

### 3.3. Comparative Quantification of Microcystis aeruginosa with Previously Established Method

**Figure 5:**
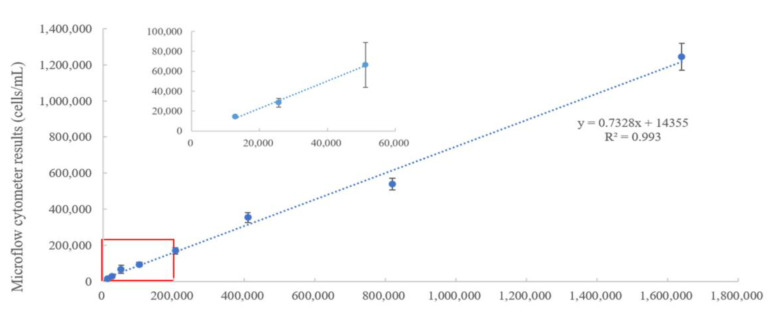
Quantitative detection of M. aeruginosa measured by a hemocytometer under a microscope and microflow cytometer platform. The enlarged view shows the red area of the linear regression of cell concentrations lower than 200,000 cells/mL with error bars of standard deviations.

Hemocytometry is an established quantitative method that can be used in conjunction with a microscope to count the number of individual *M. aeruginosa* cells in a given area, to make an approximation of the total concentration. This well-established method was compared to the quantification of *Microcystis aeruginosa* by microflow cytometry, shown in Figure 5, with the (y-axis) values of each sample plotted in relation to the hemocytometer (x-axis) results. Numerous samples, spanning a large concentration range of *M. aeruginosa* (15,000 cells/mL to more than 1,000,000 cells/mL), were measured in triplicates by each method. The linearity (R^2^ = 0.993), shown in Figure 5, demonstrates the comparability of the microflow cytometer results and the hemocytometer results across a wide range of *M. aeruginosa* concentrations. Interestingly, the lower limit of the hemocytometer is at least 10,000 cells/L; thus, the lowest cell concentration that would be sufficiently valid for this comparison should be around 15,000 cells/mL. Given that the microflow cytometer can be validated for accuracy at concentrations of roughly 15,000 cells/mL, this would suggest that the microflow cytometer is capable of detecting cyanobacterial concentrations below the WHO Alert Level 2 threshold of 100,000 cells/mL. Therefore, this technology can alert researchers and public service workers of potentially hazardous concentrations of algal blooms, far in advance of the WHO Alert Level 2 standard. This reliable and timely cyanobacterial monitoring tool can be used to analyze recreational water to better protect the public from potentially hazardous cyanobacterial blooms greater than 20,000 cells/mL [16,26,27]. Given this limit, a pre-filtration ACCRS step may be used to improve the microflow cytometer detection limits to levels comparable to the WHO Alert Level 1.

### 3.4. Low-Level Cyanobacterial Quantification towards Development of Early Warning Alerts

The ACCRS can be used to lower the detection limit of the microflow cytometry platform beyond its non-ACCRS limit of ~20,000 cells/mL. With the ACCRS, on-site water samples of roughly 1 L can be concentrated into a retentate of volumes between 1–2 mL, which will further facilitate its inclusion in the microflow cytometer process. To do so, on-site water samples containing *M. aeruginosa* were serially diluted, in a 10-fold serial dilution, to achieve multiple sample concentrations of (4 × 10^7^ cells/mL), shown in Table 2. These diluted samples were then processed and quantified using both the microflow cytometer and the hemocytometer for more accurate concentrations. Three original water samples labelled A, B, and C were quantified to determine their respective cell concentrations of 429,000 ± 5131 cells/mL, 38,800 ± 1257 cells/mL, and 4470 ± 46 cells/mL. Afterwards, 1 mL from each of these original samples were spiked into 1 L PBS to make retentate sample versions of A, B, and C. The retentate samples were added to the recovery system, with a cleaning process in between each sample test to minimize cross-contamination and were quantified with the microflow cytometer. Unfortunately, due to the variance in retentate volumes caused by the vibrations of the peristaltic pump, the range of retentate volumes spanned 1.1–1.5 mL. The high concentration ratios and milliliter scale retentate reveal that the additional ACCRS is capable of processing large amounts of water samples for microfluidic devices as a pre-filtration process.

The recovery efficiency is defined as the ratio of the total cell abundance retained in the final retentate to the cell abundance in the initial sample, expressed as a percentage. The equation is as follows:

$$\mathrm{Recovery}\;\mathrm{efficiency}\;\left(\%\right)=\frac{\mathrm{Concentration}\;\mathrm{After}\;\mathrm{Filtration}\times \;\mathrm{Retentate}\;\mathrm{Volume}\;\left(\mathrm{mL}\right)}{\mathrm{Concentration}\;\mathrm{Prior}\;\mathrm{to}\;\mathrm{Filtration}*1000\;\mathrm{mL}\;\left(\mathrm{for}\;\mathrm{original}\;1L\;\mathrm{sample}\;\mathrm{size}\right)}*100$$

With recovery efficiencies averaging well above 85%, as seen in Table 2, the additional ACCRS step demonstrated its significance to the quantification and detection processing of cyanobacterial samples. The basic equation provided above demonstrates the formula used to calculate each individual replicate’s respective recovery efficiency. The recovery efficiencies shown in Table 2, being at least 85%, demonstrated that no significant amount of analyte was lost throughout the process and was, in part, due to the effectiveness of the ACCRS. However, the calculated recovery efficiencies for samples A and C were shown to be over 100%. This may initially appear to be an improper value, but given the calculated uncertainties respective to each sample, they fall within the 100% theoretical maximum recovery efficiency. More importantly, this effectiveness was clearly seen across the large range of cyanobacterial cell concentrations from 4.5–429 cells/mL. In addition, with a detection limit of roughly 5 cells/mL and recovery efficiency of above 85%, the combination of ACCRS and microflow cytometry proves its ability to significantly improve the limit of detection compared to a microflow cytometer alone. In other words, these results indicate that the microflow cytometer platform is capable of monitoring low concentration cyanobacterial samples with high accuracy and can be used to evaluate large amounts of water samples in a very short period. The increased limit of detection in the ACCRS-microflow cytometer platform is well below the standardized cyanobacterial concentration WHO Alert Level 1 (2000 cells/mL). Therefore, it is evident that the microflow cytometry platform can be used for early waring alerts for potential harmful cyanobacterial blooms at a large range of cyanobacterial concentrations.

This technology can be further applied to better analyze the growth cycle of cyanobacterial blooms, in the hopes of characterizing trends that may serve as effective predictors of potentially hazardous bloom formations. A.J. van der Westhuizen et al. reported that the best doubling time (t_d_) for *Microcystis aeruginosa* was 1.23 days at 32 °C, and the maximum time period increased to 6.79 days at 16 °C [28]. It was observed that as the environmental temperature surrounding the cyanobacteria during growth is inversely related to the growth rate, the corresponding growth rates (k = ln 2/t_d_) are 0.56 day^−1^ at 32 °C and 0.10 day^−1^ at 16 °C, respectively. Assuming every cyanobacterial cell will double in number during every cell cycle, and if the degradation rate of the cells can be ignored, the proliferation of cells can be determined by the following equation:

N_t_ = N_0_ * 2^kt^

where k (day^−1^) is the growth rate, N_t_ (cells/mL) the cell concentration at time t (day), N_0_ (cells/mL) the initial cyanobacterial cell concentration, and t (days) the time [29,30]. Based on this growth model, by solving for t (days of growth), it takes 15.43 days (at 32 °C) and 86.44 days (at 16 °C) for *Microcystis aeruginosa* to grow from 5 cells/mL (N_0_) to 2000 cells/mL (N_t_). Given that the calculation is based on maximum growth rate, and the decay is ignored, the expected bloom time in a real situation will be longer than the calculated time periods. Therefore, there is an opportunity to further explore the theoretical growth rate versus the real-time growth rate of these algal blooms by implementing this technology in the field. Currently, with our given limit of detection, we can identify cyanobacteria in concentrations of 5 cells/mL and observe their growth in real time. This can be applied to observe the growth of cyanobacteria in each body of water and used to identify when the concentration of cyanobacteria has reached a potentially harmful threshold of at least 1000 cells/mL, which is still twice as early as the minimum WHO Alert Level 1 of 2000 cells/mL. The temperature of the water, on each day, should also be accounted for, to better determine the potential time before algal bloom development.

Besides the low-level warning alert, large volumes (>1 L) of low-level microbial samples have been effectively treated by the pre-filtration process to make them suitable for microfluidic devices. Microfluidic devices manipulate samples on a micron- or nano-scale, making it difficult and time-consuming for them to capture the entire cell abundance on a liter scale. Moreover, the limit of detection of the microfluidic device can be significantly reduced with the pre-filtration process.

## 4. Conclusions

In this study, a microflow cytometry platform was designed for the early detection and quantification of cyanobacterial in various bodies of water to reduce the potential hazards to humans caused by cyanotoxins from cyanobacterial blooms. First, the in vivo fluorescence detection capabilities of phycocyanin for cyanobacteria was studied for its selectivity and detection limit. The fluorescent signal of every single cell was measured, and the transit time and threshold were extracted to distinguish positive signals from background noise. Thus, a time-domain filtering and an intensity threshold were applied to sufficiently improve the accuracy of cyanobacterial detection. With an improved linear correlation between the fluorescence and cyanobacterial cell concentration, results from the microflow cytometer showed good consistency and improved reproducibility compared to the traditional hemocytometer method (R^2^ > 0.99). The measured quantification limit of the microflow cytometer was 15,000 cells/mL and is capable of accurately monitoring cyanobacterial cells for WHO Alert Level 2. Currently, it can provide health impairment alerts to protect the public from cyanotoxins in recreational waters.

Second, an ACCRS was designed for the facilitation of low concentration cyanobacterial samples. The limit of detection of this platform was lowered from a limit of detection of 10,000 cells/mL to 5 cyanobacterial cells/mL through the use of our ACCRS concentrator. This facilitates our potential for the characterization of early-stage algal bloom formation at a limit of detection far below the WHO Alert Level 1 minimum of 1000 cells/mL and can serve as a powerful tool in future studies to better predict, and alert for, algal blooms. Moreover, the microbial sample can be reduced from a liter scale to a 1 mL scale by the pre-concentrating system and is suitable for other microfluidic devices. Thus, this platform can also be applied to microfluidics that measure large volume environmental samples of low-level microbial targets.

Lastly, for future iterations and research studies, utilizing this platform may benefit from statistical analysis done on the dependency of this platforms efficacy to flow rates and low detection limits. By performing these statistical analyses, the overall performance, robustness and significance of data collected by this system may be further enhanced, adding to its novelty and on-site potential.

## Figures and Tables

**Table 1:** Reproducibility of the microflow cytometer depicting the number of replicates (*n*), standard deviation (SD), and coefficient of variation (CV).

Sample	Mean (Cells/mL)	SD (Cells/mL)	CV (%)	*n*
1	1,240,000	74,700	6.01	15
2	590,000	22,500	3.82	15
3	354,000	27,300	7.82	15
4	169,000	16,600	9.81	15
5	103,000	9400	9.08	15
6	56,800	15,700	27.58	15
7	28,400	4240	14.92	15
8	14,400	1360	9.48	15

**Table 2:** Determination results of low-level cyanobacterial samples after filtration. Recovery efficiencies were calculated using total derivate equation-based standard deviations and means.

Sample	Concentration Prior to Filtration (Cells/mL)	Concentration After Filtration (Cells/mL)	Volume of Post-filtration Sample (mL)	Recovery Efficiency (%)
A	429 ± 5.1	394,000 ± 1154	1.1	101 ± 1
B	38.8 ± 1.3	22,100 ± 153	1.5	85 ± 2
C	4.5 ± 0.5	3050 ± 540	1.5	102 ± 17

**Figure A1:**
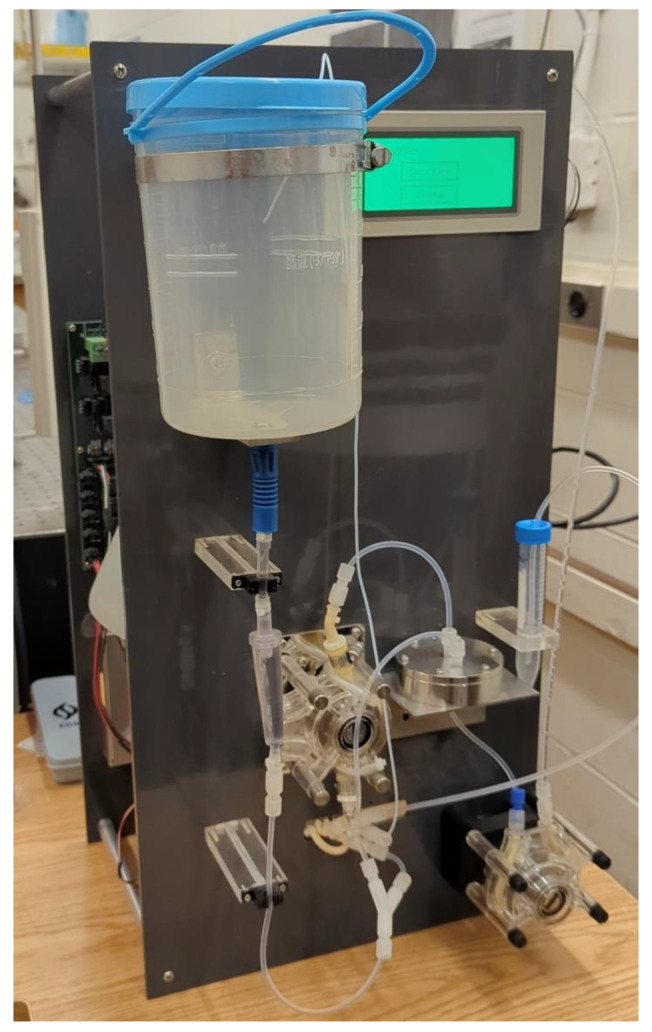
The image of the upgraded ACCRS, with integrated pre-programmed concentration and cleaning protocols, to facilitate its use for on-site detection and analysis of cyanobacteria.

## Data Availability

Data may be available upon request.
